# Analecta

**Published:** 1842-03-19

**Authors:** 


### ANALECTA.

*On Gangrenous Affections in the Puerperal State.*—The mode in which gangrene takes place, and the causes which give rise to it, are often exceedingly obscure, except indeed in those cases in which it can be attributed to a direct obstruction of the circulation, as from closure or disease of the bloodvessels, or from mechanical constriction of any of the tissues of the body. Formerly the difficulty was imagined to be at once explained by summarily saying that gangrene is one of the consequences of inflammation. In the present day, on the contrary, some authors have gone so far to the opposite extreme as to think that, even in those cases where inflammation precedes the occurrence of the death of a part, this latter lesion is not the necessary effect of the former; but that it is connected with it accidentally, either from some obstruction to the course of the blood produced indeed by the inflammatory action, or from the introduction into the system of some foreign substance, such as air or some poisonous matter, or lastly, from a peculiar morbid state of the constitution itself.

Struck with the frequency with which gangrene takes place in the course of puerperal diseases, M. *Reynaud*, in his recently published memoir on this subject, has brought together many cases in illustration, and after pointing out the fluctuating and ill-supported opinions of most writers—opinions which are almost always based on the doctrine of gangrene being the result of inflammation or of putridity—he groups them in two classes:—1, gangrenous affections attributable to inflammation, and 2, those attributable to infection.

As to the cases reported in the first class, we might have expected to find that in them there had been present all, or at least the most prominent, signs of inflammatory action, developing itself gradually with greater or less rapidity, and ultimately terminating in gangrene; and yet this is not the case, if we may judge from the details narrated in the reports. Thus, in the first case, which is headed *puerperal peritonitis with gangrene of the abdominal parietes, peritoneum, and intestine*, the gangrene appeared, on the eleventh day after accouchement, on the surface of the abdomen, without having been preceded by any redness or swelling of the part; and it is not stated in the dissection of the patient, who died at the end of the third week, that the gangrenous portion of the abdominal parietes exhibited any traces of pre-existing inflammation.

The author narrates among the same group several cases where death occurred a few days after laborious delivery, and in which the internal surface of the uterus exhibited a gangrenous eschar of some lines in thickness. In these cases he attributes the lesion to the violent manœuvres which the body of the womb had sustained. But, when the uterus has been in part disorganised by the efforts which a difficult labour has rendered necessary, should we attribute the gangrene in such cases to inflammatory action? Is it not rather a result altogether mechanical, such as we observe to take place after severe contusions, in which inflammation has little or no part at all?

The author seems to have been himself aware how little the phenomena of inflammation can be supposed in such cases to have any thing to do with the development of gangrene; as he tells us that, in these circumstances, we must not forget to take into account the condition of the female system after labour, or, in other words, the puerperal state. On the whole, we are inclined to give it as our opinion, judging from the very cases which M. ***Reynaud*** has recorded in his first class, that inflammation has only a very doubtful part in the production of gangrene in puerperal women; at the same time we acknowledge that it often accompanies it, as if the two states, gangrene and inflammation, were the result of the same cause. This leads us to the consideration of the *second* set of cases, those of gangrenous affections induced by infection.

Puerperal diseases arising from infection differ from diseases attributable to the same cause in this particular, that in the latter the infection usually proceeds from without, while in the former it is engendered in the bodies of the patients themselves: such is at least the opinion of the physicians of the French school, who entirely reject the doctrine of the transmission of puerperal affections by direct contact.

Whatever may be the origin of the infection, we must not the less keep in remembrance that, occasionally under the influence of certain changes in the fluids of the body, we observe gangrene taking place. It is true that if we try to push our enquiries further, and to discover what it is that constitutes infection, or how it acts in producing gangrene, we find ourselves quite at a loss; but it is one step, and that an important one, gained, to have ascertained that such cases are of too frequent occurrence to be regarded as merely the results of a simple coincidence.

In this point of view, the work of M. *Reynaud* is very interesting, although many of his observations are far from being satisfactory. What connexion, for example, can be found between the external veins filled with pus, and pulmonary gangrene, or between even inflammation of the lymphatic glands and gangrene of the uterus? None, certainly, as far as we can trace; and yet we do not deny positively that these different morbid states may not be connected with each other. We are disposed to attach more importance to another source in explaining some of the facts adduced by M. ***Reynaud***, in illustration of the tendency in the female system after delivery to gangrenous affections—we mean, the absorption of unhealthy secretions from the internal surface of the womb, and their introduction into the circulation, first of the uterus, and ultimately of the entire system.

The ingenious explanation given by M. *Genest* of the development of pulmonary gangrene in cases of apoplexy of the lungs and of metastatic abscesses in their parenchymatous tissue, by the admixture of the external air with the blood or purulent matter, should certainly not be lost sight of in investigating the phenomena of certain puerperal diseases.

We are glad to observe, from a recent account of the different forms of puerperal fever observed at the Hôtel Dieu in Paris, during the year 1840, by M. *Bourdon*, that this intelligent physician has shaken off the trammels of the Broussaian school as to the proximate cause of the disease:

He says, "The fever exhibited always the same characteristic symptoms, with a peculiar aspect and march truly remarkable; while the lesions found on dissection were very different in different cases. Several of the lesions might be attributed to the inflammation, which, in this disease, terminates with extraordinary rapidity and ease in the formation of purulent matter in different parts of the body, such as the posterior parts of the limbs, the sub-peritoneal cellular tissue, &c.; but we could not regard certain other serious lesions, as the consequence of this peculiar kind of inflammation."

If, at the same time, we call to mind the fluid state of the blood in the heart and large bloodvessels, the softening of almost all the viscera, &c. are we not authorised to regard puerperal fever as a disease which is intimately connected with some change or poisoning of the blood?—*London Medico-Chirurgical Review*, January, 1842, from *Gazette Medicale.*

*Remarks.*—With many of the preceding observations we heartily concur; and rejoice to find that, in France, as well as in our own country, some of the most practical writers of the day are beginning to pay attention to the state of the fluids in disease, more especially in malignant fevers. The doctrine that puerperal fever is in all, or even in most, cases an essentially inflammatory disease, and, therefore, that it requires an active antiphlogistic treatment, is fraught with the most pernicious consequences. The capital error that has been committed by most writers arises from the opinion that this fever is at all times and in all seasons of the same *type;* whereas, in truth, perhaps no two epidemics of the disease are alike; just in the same manner as the epidemics of typhous and of the exanthematous fevers are observed to vary exceedingly in different years. We have too long forgotten to take into our consideration of such diseases the influence of what *Sydenham* and many of the old writers have denominated the *medical constitution of the season;* and yet, what practical physician can have failed to observe the striking difference in the general character, as well as in the mortality, of febrile disorders in different seasons? Take, for example, scarlatina; is it not sometimes so mild as scarcely to require any medical treatment at all? whereas during another year it is attended with high phlogistic symptoms, and in a third it exhibits the type of malignancy and putridity.

The same holds good of puerperal fever. It is quite true that in some epidemics, and, we may add, in some cases during all epidemics, the disease is essentially inflammatory; but it is equally true that in other cases the inflammatory symptoms, if they occur at all, are only added to, and, as it were, grafted upon a morbid state of the system, which is dependent upon a vitiated state of the fluids, and a consequent serious lesion of the entire nervous system. Now this is very nearly the doctrine of the older school; and, however humiliating to modern pride it may be to find that with all our improved methods of research we are in a great measure coming back to the long obsolete opinions and practice of the last century, it is only wise and right that we should do so when convinced of our errors.

We cannot close these few remarks without recommending to the especial notice of our readers the admirable Treatise on Puerperal Fever by Dr. *Ferguson*, published two years ago, and of which a copious review was given in our number for April 1839. One very short extract will enable them to judge of the spirit of its contents.

"The three following propositions embody my views of the source and nature of puerperal fever.

1. The phenomena of puerperal fever originate in a vitiation of the fluids.
2. The causes which are capable of vitiating the fluids, are particularly rife after child-birth.
3. The various forms of puerperal fever depend on this one cause, and may readily be deduced from it."—(*Rev.*)

---

*Severe pain in the tibia relieved by incision.* By CHARLES JAMES FREEMAN, Surgeon.—Benjamin Kirby, thirty-eight years of age, complained on the night of Thursday, July 29th, of pain down the shin bone, which increased to such a degree as to induce him to send for medical advice. Upon questioning him, he described the pain "as if something alive was at the bone; but on examination there was neither swelling, redness, or tension of the skin apparent; the pain was confined to the middle portion of the anterior surface of the tibia, and was constant, though more severe at stated periods, about every six hours. This pain was accompanied with high fever; pulse hard, full, and 120; he was bled generally and locally, and salines with mercurials were administered. These measures not removing either pain or febrile symptoms, I ordered a repetition of leeches, with emollient poultices and tartarised antimony: these afforded no relief, and I had recourse to large and continued doses of mercury, but with as little success. I found hectic symptoms coming on; stomach rejected all food; no sleep; opiates useless; flushes succeeded by cold cadaverous palpitations; still there were no external marks denoting inflammation. However, I was convinced relief must speedily be afforded, or the patient would sink, and therefore hazarded an incision, which I carried down the anterior surface of the tibia to the extent of six inches, and divided the periosteum; I applied a large bread poultice to the wound, and gave gentle aperients and alteratives. The next day I saw him, he was an altered man and exclaimed, "Oh, I have been in Heaven since you cut my leg." On inquiry I found that he fell asleep within an hour after the incision had been made, and slept for six hours. From that time he gradually improved under a generous diet, nor had he any return of pain. At the end of a fortnight he was sufficiently recovered to attend to his occupation of lime-burning. The wound healed without any trouble, and he is in perfect health, the leg being equally strong with its fellow.—*London Lancet*, *Nov.* 13, 1841.

---

*The Vascularity of Tubercle.* By P. N. KINGSTON, M. D.—The analysis which appeared in your journal on September 24th of a lecture by M. Lugol—a high authority on the subject in question—affords ample confirmation of the observations I published in 1836, on the organization and vascularity of tubercle. It is stated that M. Lugol has, in numerous cases, detected blood-vessels ramifying, not only in the cyst, but in the tubercular matter itself. In my paper on Tubercle, published in the twentieth volume of the Transactions of the Royal Medical and Chirurgical Society of London, I gave an account of seven cases in which great numbers of pulmonary tubercles, of the ordinary kind, presented, under the microscope, red vessels, which ramified and anastomosed through their interior, and were connected at the circumference with the vessels of the lungs; and I mentioned that in one of these cases, red vessels were also seen in tubercles of the bronchial and mesenteric glands.

I may add, that five months after my paper was laid before the Society, Dr. James Macartney, the distinguished Professor of Anatomy at Trinity College, Dublin, stated at the British Association, that he had suceeeded in injecting tubercle.

The concurrent testimony of observers, who appear to be ignorant of one another's observations, will, I trust, be considered by your readers as demonstrative of the fact: and as these vessels are only now and then visible, and cannot be with certainty discriminated without much patient examination with the microscope, they will not be disheartened, if they should not in their first attempts succeed in detecting them.

Another instance in which a texture, long supposed to be unorganised, has now been proved to possess blood-vessels, is furnished by articular cartilage in the adult. Its vessels, being in health very minute, and not destined to transmit red blood, were not easily detected, even after careful attempts at injection; and were, in consequence, positively denied to exist by Cruveilhier, Velpeau, Key, and others. Nevertheless, Bichat, Gordon, Brodie, Mayo, and Alison, saw reason confidently to infer that they must be vascular and organized. Bichat noticed that they were liable to be reddened by inflammation; and Sir Benjamin Brodie met with occasional, though rare instances, of vessels containing red blood extending from a diseased bone into the cartilage covering it. At last Mr. Liston has shown that the injection of articular cartilage, which had been generally maintained to be impossible, from a total absence of vessels, is only difficult, and may with skill be, under favourable circumstances, accomplished.—*Lond. Med. Gaz. Nov* 26, 1841.

---

*On the Regeneration and Union of Nerves.* By MM. Gunther and Schon.—Rabbits were the animals on which the following observations were made, and about fifty of these were operated on, by sometimes only cutting across the ischiatic nerves, at other times by removing a portion varying from two to four lines in length. These physiologists describe the elementary fibres of the nerves as transparent cylinders with double tunics, filled with a fluid resembling liquid albumen. After maceration in water, or after death, this fluid coagulates and produces the turbid granular aspect which, till now, has been considered its natural appearance. When the two extremeties of a nerve which has been cut across become united, the nerve propagates impression through the uniting medium. This is done by means of true primary fibres having been formed through the uniting medium, and the following is the mode in which MM. Gunther and Schon have observed this to take place. After division of the nerve the two ends retract somewhat, the diameter of the neurilema becomes diminished, and the medullary substance is pushed out in a globular form; exudation of plastic lymph then occurs and fills the wound, and the cut extremities of the nerve become swollen, the upper extremity more than the lower. This tumefaction of the nerve itself is owing to the presence of plastic lymph effused into the cellular tissue and and also between the neurilema and primative fibres.

The matter which unites the two ends of the nerve, is at first amorphous, but by degrees primitive fibres shoot through the mass, and become visible at the earliest on the eighth week after division. These new fibres are in every respect similar to the original fibres; but the granular exudation and the cellular tissue which surround and envelope them render them difficult of examination. These fibres are not parallel, but exhibit a confused arrangement.

With the regeneration of the fibres of the nerve returns the sensibility and mobility of the limb or organs to which the nerve was distributed.

But in general the function of the part is not so free as before the division; the animals not being able to use the limb whose nerve had been divided, so freely as the other. The influence of the will over the limb was also observed to be diminished. MM. Gunther and Schon account for this on the supposition, which is borne out by their experiments, that the number of fibres which are regenerated are not equal to those which originally existed; besides it appeared to them that the regenerated fibres occasionally united the ends of different primitive fibres, so that sensation was not always referred to its proper place. They consider that the regeneration or growth of the uniting fibre commenced at the superior extremity of the divided nerve, but concede that it is possible that it may also take place from the lower extremity.—***Edinburgh Med. and Surg. Journ. from Arc. Gen. de Med., March***, 1841.

---

*Experimental Researches on the Function of the Skin in man and animals.* By Dr. Ducros.—In a very curious experimental paper, Dr. Ducros shows that a coating of gum-lac put on the skins of animals causes them to die in a longer or shorter time, by producing convulsive movements similar to epilepsy. When the animals, coated with gum-lac, were subjected to electricity, they died in a much shorter time. He next tried the effect of metallic coverings, as he entertained the notion that, because they had opposite electrical properties, the animals coated with them would die with symptoms of an opposite nature. He therefore cut off the hair from some animals and covered them with thin plates of tin, (tin-foil) and found that they perished with symptoms of debility, the reverse of what he had noticed when the coating consisted of a resinous substance. When the tin was covered with a coating of gum-lac, the animals perished much more rapidly. He then placed under the influence of electricity some of the animals covered with plates of tin, and found that so long as they remained connected with the electrical current, their vigour appeared to be restored, but that whenever it was arrested, they appeared ready to perish.

The object of these experiments was to ascertain what would be the likely effect of such coverings in certain diseased states of the human frame, and especially in nervous or neuralgic affections and in rheumatism. He reasoned, that, if metallic coverings deprived animals of life by producing rapid sinking of the vital powers, the same metallic plates applied to the human body would cure or remove those diseases which seemed to depend on an excess of organic life. On putting his plan to the test of practice, he was so fortunate as to find that it removed some nervous, and a few acute and chronic rheumatic affections.

This plan of treatment was of no avail in any case where the diease was dependent on or connected with organic lesions, or attended with fever, or swelling of the part, or with general weakness; on the contrary, in all these cases the metallic plates augmented the disorder.—***Ibid. from Comptes Rendus des Seances de l'Acad. des Sciences, 20th Sept.*** 1841.

---

*Mode of Preventing the formation of Gouty Concretions.* By Alexander Ure, M. D.—The urate of soda, which forms the chief bulk of gouty concretions, being an extremely insoluble salt, Dr. Ure thought that if any means could be devised, so to modify the secretion of the urates, so as either to render them soluble, or to supersede them altogether, these concretions might be prevented from forming. In the course of his researches, he found that hippuric acid combined with soda, which he regarded as the analogue of gouty concretions, occurred in the urine of graminivorous animals. This salt is extremely soluble, two parts of water at 60° dissolving one part of it. Dr. Ure then ascertained by repeated experiment on himself and others, that the one salt might be substituted for the other in the human body, without any risk of affecting the general health or irritating the urinary organs. This was accomplished by administering internally the benzoic acid, an hour after a meal, when he found that the urine voided a couple of hours afterwards gave on the addition of muriatic acid, a copious precipitate of beautiful rose-pink acicular crystals of hippuric acid. The quantity procured in this way was, in general, equivalent by atomic computation to half of the benzoic acid employed, so that the remainder of that acid must have made its escape by some other emunctory. The same result was obtained when the hippurates of potash or of ammonia were used, and he thinks it would often be found preferable to employ them instead of the simple acid. The dose could easily be apportioned to the state of the urinary secretion, previously ascertained by experiment. Thus, by a process of vital chemistry, an acid containing 8 atoms of azote and 10 of carbon, is replaced by one containing 18 of carbon and only 2 of azote, and that too in what has been regarded as a highly azotized state of the system.

Mr. Ure has not yet ascertained how far this plan of treatment is applicable to all the various forms of calculous diseases connected with gouty diathesis; but he has not found it to interfere with the other remedial means; and unequivocal proofs, he says have been already afforded him, of its efficacy in correcting and removing certain disordered states of the urine in individuals prone to attacks of gravel.—*Ibid. from Medico-Chirurgical Trans.* 1841.

